# GDSL in *Lilium pumilum* (LpGDSL) Confers Saline–Alkali Resistance to the Plant by Enhancing the Lignin Content and Balancing the ROS

**DOI:** 10.3390/ijms25179319

**Published:** 2024-08-28

**Authors:** Zongying Wang, Wenhao Wan, Miaoxin Shi, Shangwei Ji, Ling Zhang, Xiaolu Wang, Lingshu Zhang, Huitao Cui, Xingyu Liu, Hao Sun, Fengshan Yang, Shumei Jin

**Affiliations:** 1 Key Laboratory of Saline–Alkali Vegetation Ecology Restoration, Ministry of Education, College of Life Sciences, Northeast Forestry University, Harbin 150000, China; 2 Engineering Research Center of Agricultural Microbiology Technology, Ministry of Education, Heilongjiang University, Harbin 150080, China; 3 Heilongjiang Provincial Key Laboratory of Ecological Restoration and Resource Utilization for Cold Region, Heilongjiang University, Harbin 150080, China; 4 Key Laboratory of Molecular Biology, College of Heilongjiang Province, College of Life Sciences, Heilongjiang University, Harbin 150080, China

**Keywords:** *GDSL* gene, *Lilium pumilum*, lignin, ROS, saline–alkali stress

## Abstract

In order to explore the response mechanism of *Lilium pumilum* (*L. pumilum*) to saline–alkali stress, we successfully cloned *LpGDSL* (GDSL lipase, Gly-Asp-Ser-Leu) from *L. pumilum*. The qRT-PCR results indicated that the *LpGDSL* expression was higher in the leaves of *L. pumilum*, and the expression of the *LpGDSL* reached the highest level at 12 h in leaves under 11 mM H_2_O_2_, 200 mM NaCl, 25 mM Na_2_CO_3_, and 20 mM NaHCO_3_. The bacteriophage overexpressing *LpGDSL* was more tolerant than the control under different NaHCO_3_ contents. Overexpressed and wild-type plants were analyzed for phenotype, chlorophyll content, O_2_^−^ content, H_2_O_2_ content, lignin content, and so on. Overexpressed plants had significantly higher resistance than the wild type and were less susceptible to saline–alkali stress. The yeast two-hybrid and BiFC assays demonstrated the existence of an interaction between LpGDSL and LpBCP. The yeast one-hybrid assay and transcriptional activation assay confirmed that B3 transcription factors could act on *LpGDSL* promoters. Under saline–alkali stress, *L. pumilum* will promote the expression of *LpGDSL*, which will then promotes the accumulation of lignin and the scavenging of reactive oxygen species (ROS) to reduce its damage, thus improving the saline–alkali resistance of the plant.

## 1. Introduction

Salt and alkali stress has restricted the growth and development of plants. Therefore, the identification of saline–alkali tolerance genes and related studies are of great importance for the effective use of barren land.

*L. pumilum* is characterized by salinity and drought tolerance, which can be used as an important parental resource for breeding resistance in lily species [1].

The conserved structural domain of the GDSL protein was first characterized and the lipase was named by Upton and Buckley in 1995 [2]. The GELP family contains many functional genes that have important biological functions in plant growth and development, morphogenesis, and defense responses. GDSLs are localized in the extracellular matrix, chloroplasts, cytoplasm, nucleus, vacuole, and endoplasmic reticulum [3]. GDSL lipase 1 (*AtGLIp1*) was highly expressed in the seedlings, leaves, and roots of *Arabidopsis thaliana*, as well as in stems and flowers, while *AtGLIP2* was only expressed in seedlings, stems, and roots, all of which were associated with disease resistance [4,5]. The expression of *GDSL* can be influenced by hormones, chemicals, and environmental stress [5,6]. The *CpGLlP1* gene promoter contains elements related to plant abiotic threats such as LTR, STRE, MYC, and MYB, and hormone-induced elements such as CGTCA-motivated and ABRE [7]. Rice GSDL family members were expressed under treatments such as chromium, arsenic, cadmium, and lead, as well as hormones, oxygen deprivation, drought and waterlogging stress, and high-salt and high-heat treatments [8]. Yeast strains with heterogeneous *AtGELp60* have good tolerance to LiCl stress [9]. In *A. thaliana*, the GDSL esterase/lipase family is involved in the regulation of plant cell wall components [10]. 

In terms of abiotic stress, the proteomic analysis of sugarcane seedlings under salt treatment by Chiconato et al. found that the expression of GDSL lipase in a salt-treated group of tolerant varieties was three times higher than that of the control group [11]. Hong et al. found that the expression of chili GDSL lipase was induced by various stress factors such as drought and high salt [12]. Zhao et al. analyzed the proteome of oat seedlings under alkali stress and found that a large number of resistance proteins, such as GDSL lipase, had accumulated in the roots of oat resistant to alkali stress [13]. After 20 mM NaHCO_3_ treatment, the transcriptome sequencing of *L. pumilum* bulbs showed that the expression of GDSL was significantly increased compared with that of untreated plants. In summary, we hypothesize that GDSL is associated with salinity resistance in *L. pumilum*.

In this study, The *GDSL* has been cloned from *L. pumilum* and a detailed functional analysis of its function has been conducted so as to fully understand the response relationship between *LpGDSL* and saline–alkali stress.

## 2. Results

### 2.1. Cloning and Bioinformatics Analysis of LpGDSL Gene

The open reading frame of the *LpGDSL* gene was successfully cloned, and the sequencing results showed that it was 1080 bp (*LpGDSL* sequences are listed in Table S1), encoding 360 amino acids. LpGDSL had a highly conserved SGNH (a hydrolase with strictly conserved catalytic residues Ser, Gly, Asn, and His, so abbreviated as SGNH) lipase domain at the position of amino acid 300-1165 (Figure S1). The LpGDSL protein had a high homology with the GDSL protein of various plants, such as *Dendrobium catenatum*, *Phoenix dactylifera*, *Musa acuminata* subsp. *Malaccensis*, *Asparagus officinalis*, *Nelumbo nucifer*, *Phalaenopsis equestris*, *Actinidia eriantha*, and *Prosopis alba* (Figure S2). Phylogenetic tree results showed that LpGDSL was most closely related to the GDSL proteins of asparagus, jujube, and Canna (Figure S3).

### 2.2. mRNA Expression Specificity Analysis and Yeast Resistance Analysis 

**Figure 1:**
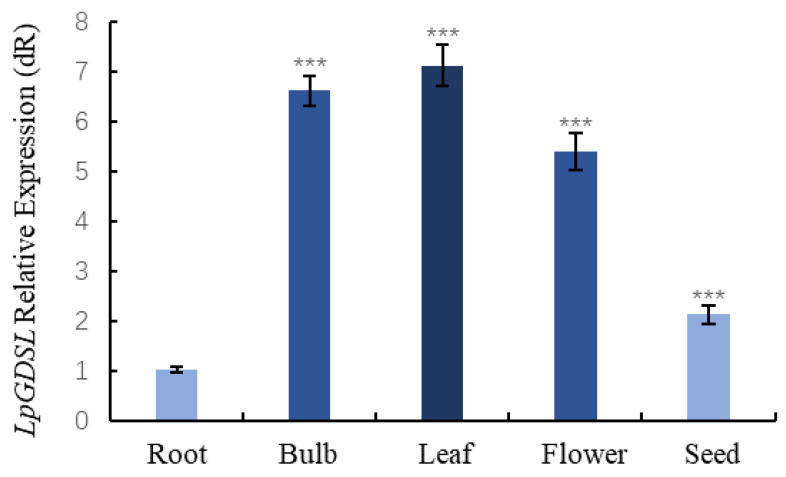
Expression of *LpGDSL* gene in different tissues of *L. pumilum*. The RNA in the different organs of *L. pumilum* (roots, bulbs, leaves, flowers, and seeds) was extracted and then reverse-transcribed into cDNA, and the levels of relative expression were determined through qRT-PCR. *** *p* < 0.001, standard error of three biological replicates.

*LpGDSL* is widely expressed in all tested tissues (Figure 1), but its expression is most abundant in the leaves, bulb, and flower. The expression level in the leaves is 7 times that in the roots and about 3 times that in the seeds. 

**Figure 2:**
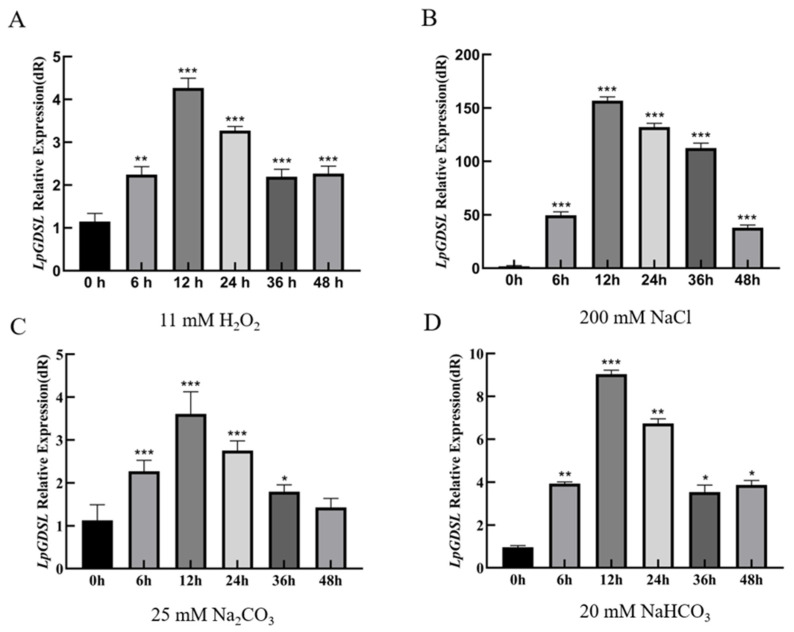
Expression of *LpGDSL* in *L. pumilum* under different salt–alkali stress. RNA was extracted from stress-treated *L. pumilum* leaves and then reverse-transcribed to cDNA, and the levels of relative expression were determined through qRT-PCR. * *p* < 0.05, ** *p* < 0.01, *** *p* < 0.001, standard error of three biological replicates. (**A**): Expression levels of *LpGDSL* gene under 11 mM H_2_O_2_ treatment at different times; (**B**): Expression levels of *LpGDSL* gene under 200 mM NaCl treatment at different times.; (**C**): Expression levels of *LpGDSL* gene under 25 mM Na_2_CO_3_ treatment at different times; (**D**): Expression levels of *LpGDSL* gene under 20 mM NaHCO_3_ treatment at different times.

The expression levels of *LpGDSL* increased significantly under a range of stress conditions, all reaching the highest expression levels at 12 h of exposure. Subsequently, the expression of *LpGDSL* decreased with the extension of treatment time (Figure 2). The expression of *LpGDSL* showed an up-regulation trend under all kinds of stress compared to the control group.

**Figure 3:**
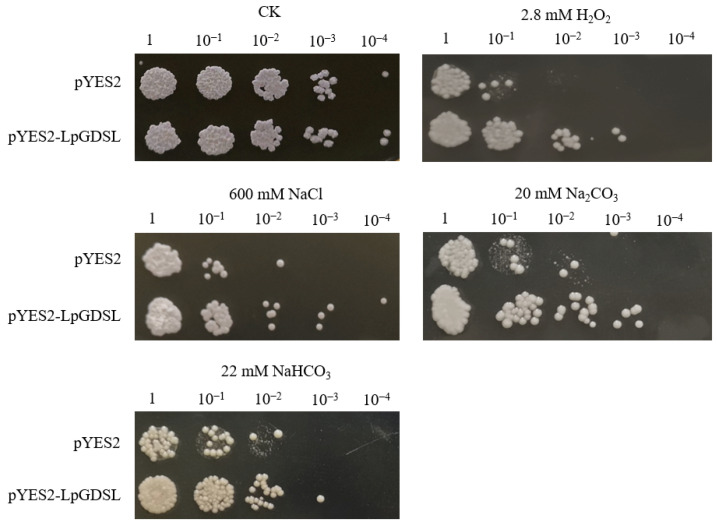
Tolerance analysis of gene overexpression in yeast strains under different stresses. The pYES2-LpGDSL recombinant yeast solution was gradient-diluted ten-fold, one-hundred-fold, one-thousand-fold, and ten-thousand-fold, and set aside (pYES2 solution was used as the control group). The concentration of the two bacterial fluids was adjusted to the same level on YPD medium without any stress, and then YPD solid medium was prepared under the stress concentrations of 2.8 mM H_2_O_2_, 600 mM NaCl, 20 mM Na_2_CO_3_, and 22 mM NaHCO_3_, and 3–4 µL of the above gradient-diluted yeast solution was vertically dropped onto the medium.

After the pYES2-LpGDSL plasmid was successfully constructed, it was transferred to the yeast stain Inscv1. Each bacterial solution with the same starting concentration (OD_600_ = 1.0) was diluted ten-fold to ten thousand-fold. In addition, the control yeast strain and recombinant yeast strains were cultured in the stress medium containing 2.8 mM H_2_O_2_, 600 mM NaCl, 20 mM Na_2_CO_3_, and 22 mM NaHCO_3_. After 3 days, it was observed that the pYES2-LpGDSL recombinant yeast grew better than the empty carrier yeast and was more tolerant to salt and alkali stress (Figure 3).

### 2.3. LpGDSL Protein Induced Expression and Purification

**Figure 4:**
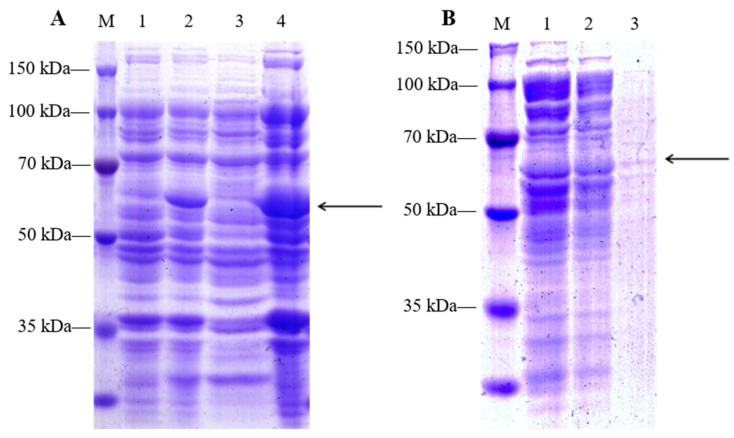
Induced expression and purification of pGEX-LpGDSL fusion protein (**A**): The optimal induction conditions for LpGDSL proteins were determined after preliminary experiments: 30 °C, 4 h, 1 mM IPTG. Induction and purification were carried out under these conditions. Massive induction of the target protein: 1: Induction for 0 h; 2: Induction for 5 h; 3: Supernatant; 4: Precipitation; (**B**): Purification of a target protein: 1: Renaturation protein 1; 2: Renaturation protein 2; 3: Purification of protein; M: Double molecular pre-staining protein marker.

**Figure 5:**
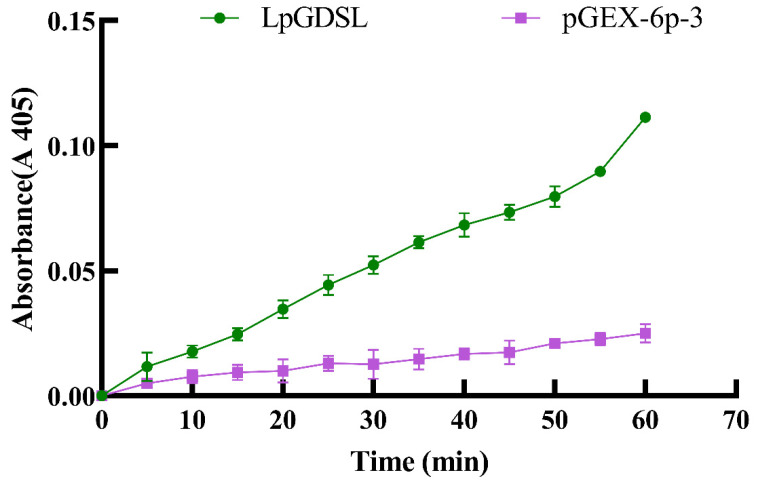
LpGDSL protease activity as compared to the control over time.

The pGEX-LpGDSL protein was successfully induced and purified (Figure 4). In order to verify the lipase activity of LpGDSL, we performed an enzyme activity assay on the LpGDSL protein. With the reaction time, the OD value of the pGEX-LpGDSL protein increased gradually at 405 absorbance compared with the purified protein of the PGX-6P-3 empty carrier (Figure 5). The p-nitrophenol product generated by the consumption of p-nitrophenylacetate by the LpGDSL protein indicated that the LpGDSL protein had the function of hydrolyzing the lipase substrate and the activity of the lipase gene.

**Figure 6:**
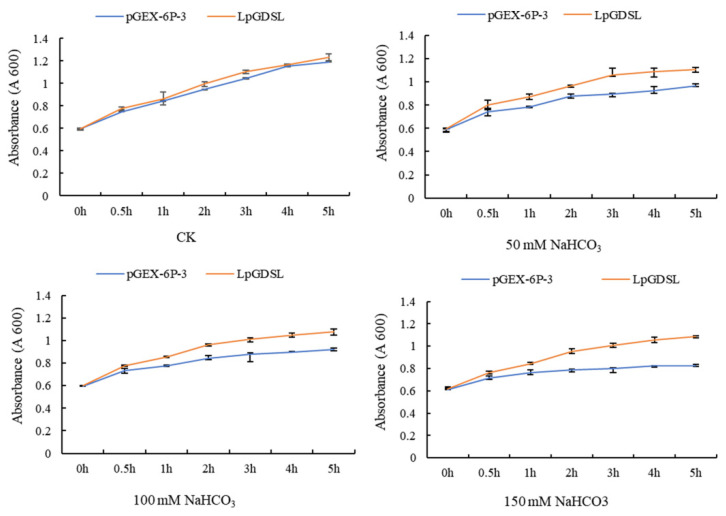
Tolerance analysis of LpGDSL protein expression strain. Protein bacteriophage resistance analysis with pGEX-6P-3 protein expression vector into BL21 receptor was used as a control group, and LpGDSL bacteriophage activity was analyzed under 0, 50 mM, 100 mM, and 150 mM NaHCO_3_ treatment.

The pGEX-6P-3 vector was transferred into BL21 bacterial solution as the control group, and the BL21 bacterial solution including pGEX-6P-3-*LpGDSL* was analyzed under the treatment of 0, 50 mM, 100 mM, and 150 mM NaHCO_3_. The activity of the pGEX-6P-3 protein-expressing bacterial solution was more strongly inhibited and became more significant with the increase in stress concentration (Figure 6). LpGDSL can promote the activity of bacterial broth and has the advantage of resistance to saline–alkali stress.

### 2.4. Resistance Analysis of LpGDSL Overexpression in L. pumilum

In order to identify whether the *LpGDSL* gene was successfully overexpressed in *L. pumilum*, leaves of wild-type and overexpressed *L. pumilum* were selected for RNA extraction and their cDNA was used as the template for real-time quantitative PCR detection. The mRNA expression levels of 1#–4# overexpressed *L*. *pumilum* were higher than that of the wild type (Figure S4). The successfully obtained *LpGDSL*-overexpressed *L. pumilum* was cultured for further study.

**Figure 7:**
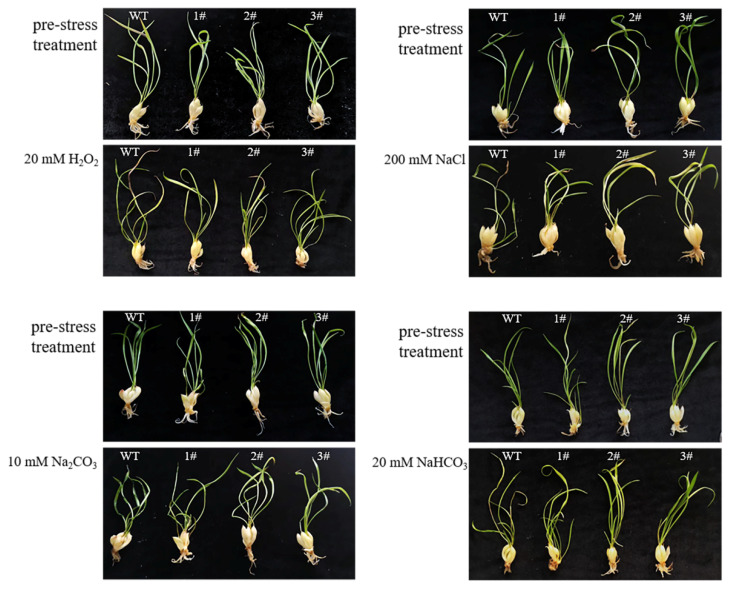
Tolerance analysis of *L. pumilum* overexpressing *LpGDSL* under stress. Phenotypic changes in wild-type and *LpGDSL* overexpression lines after 48 h in 1/2MS medium containing 20 mM H_2_O_2_, 200 mM NaCl, 10 mM Na_2_CO_3_, and 20 mM NaHCO_3_. Pre-stress: control group without stress treatment. WT: wild type. 1#, 2#, 3#: lines overexpressing *LpGDSL*.

The wild-type and *LpGDSL*-overexpressed *L. pumilum* were cultured on 1/2 MS medium containing 20 mM H_2_O_2_, 200 mM NaCl, 10 mM Na_2_CO_3_, and 20 mM NaHCO_3_. The WT leaves turned yellow, and the roots wilted after stress, while the overexpression lines could still grow normally (Figure 7). 

**Figure 8:**
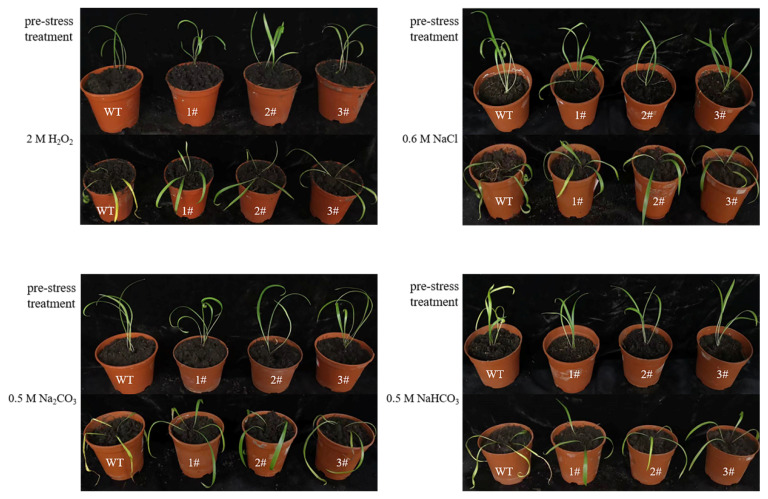
Phenotypic analysis of *L. pumilum* overexpressing *LpGDSL* under stress. Phenotypic changes in wild-type and *LpGDSL* overexpression lines after 24 h of irrigation stress with 2 M H_2_O_2_, 0.6 M NaCl, 0.5 M Na_2_CO_3_, and 0.5 M NaHCO_3_. Pre-stress: control group without stress treatment. WT: wild type. 1#, 2#, 3#: lines overexpressing *LpGDSL*.

The overexpressed *L. pumilum* had good saline resistance compared with the wild type under the 2 M H_2_O_2_, 0.6 M NaCl, 0.5 M Na_2_CO_3_, and 0.5 M NaHCO_3_ irrigation stresses (Figure 8). 

Western blot experiments were carried out on the total protein extracted from soil-cultured seedlings after each stress treatment. The LpGDSL protein expression level of the overexpressed plants was significantly higher than that of the control group (Figure S5). 

**Figure 9:**
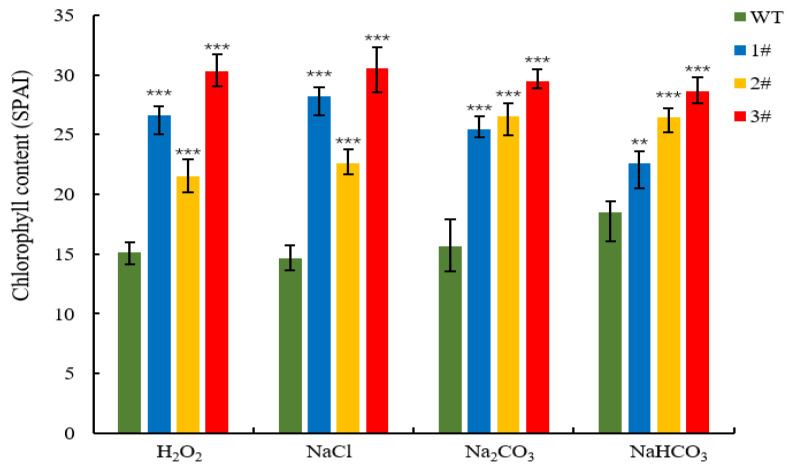
Chlorophyll content of *L. pumilum* under stress. Changes in chlorophyll content of WT and *LpGDSL* overexpression plants (1#, 2#, 3#) before and after 2 M H_2_O_2_, 0.6 M NaCl, 0.5 M Na_2_CO_3_, and 0.5 M NaHCO_3_ stress. ** *p* < 0.01, *** *p* < 0.001, standard error of three biological replicates.

**Figure 10:**
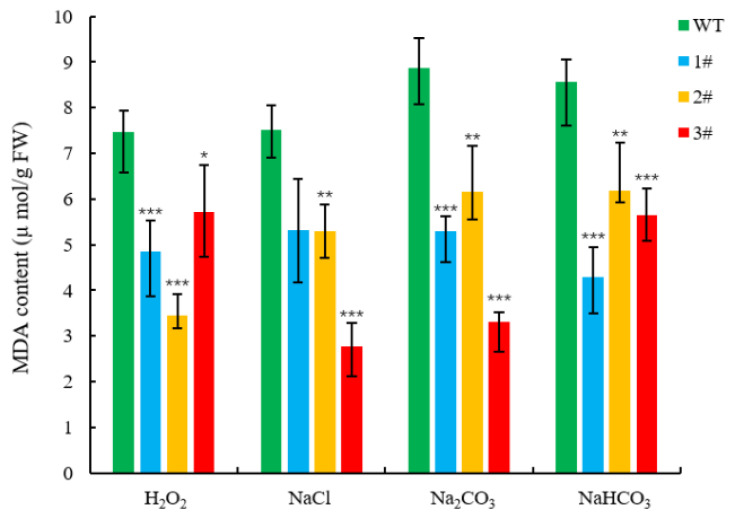
MDA content of *L. pumilum* under stress. Changes in MDA content of WT and *LpGDSL* overexpression plants (1#, 2#, 3#) before and after 2 M H_2_O_2_, 0.6 M NaCl, 0.5 M Na_2_CO_3_, and 0.5 M NaHCO_3_ stress. * *p* < 0.05, ** *p* < 0.01, *** *p* < 0.001, standard error of three biological replicates.

**Figure 11:**
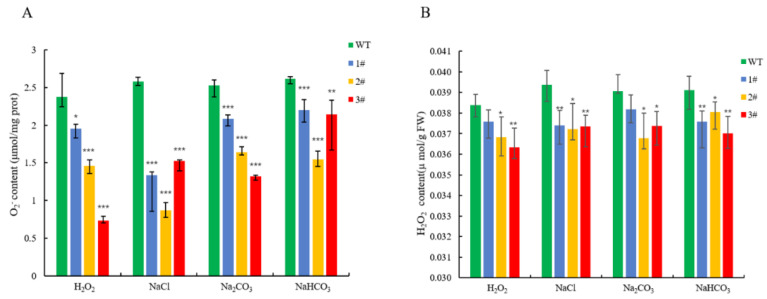
O_2_^−^ content and H_2_O_2_ content of *L. pumilum* under stress. (**A**): Changes in O_2_^−^ content of WT and *LpGDSL* overexpression plants (1#, 2#, 3#) before and after 2 M H_2_O_2_, 0.6 M NaCl, 0.5 M Na_2_CO_3_, and 0.5 M NaHCO_3_ stress. * *p* < 0.05, ** *p* < 0.01, *** *p* < 0.001, standard error of three biological replicates. (**B**): Changes in H_2_O_2_ content of WT and *LpGDSL* overexpression plants (1#, 2#, 3#) before and after 2 M H_2_O_2_, 0.6 M NaCl, 0.5 M Na_2_CO_3_, and 0.5 M NaHCO_3_ stress. * *p* < 0.05, ** *p* < 0.01, standard error of three biological replicates.

The four lines of *L. pumilum* were treated under the above concentration stresses, and the chlorophyll content of the overexpressed plants was higher than that of the wild type (Figure 9). The wild type accumulated a large amount of malondialdehyde, while the three overexpressed lines accumulated less (Figure 10). The average content of superoxide anion accumulated in wild-type *L. pumilum* after injury reached 2.5 µmol/mg prot (Figure 11A), and under NaCl and H_2_O_2_ treatment, the content was 2.08 times and 1.92 times higher than that of the overexpressed plants, respectively. The wild type was 1.31 times higher under Na_2_CO_3_ and NaHCO_3_ stress. 

**Figure 12:**
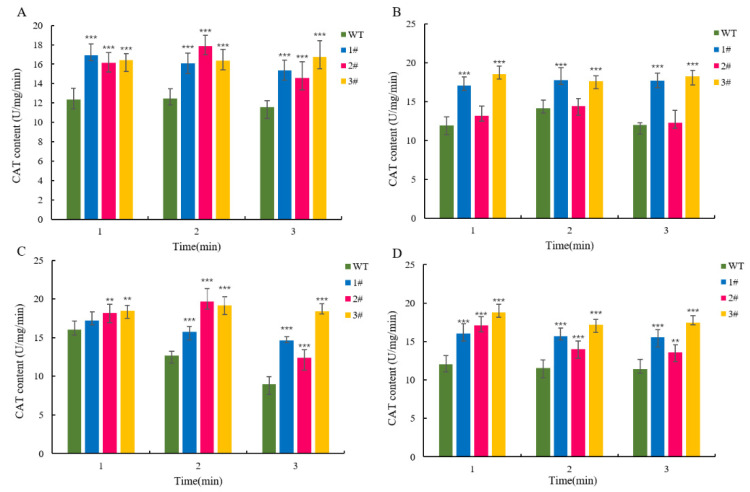
CAT content of *L. pumilum* under stress. (**A**): CAT content of wild-type and overexpression lines under 2 M H_2_O_2_ stress treatment. (**B**): CAT content of wild-type and overexpression lines under 0.6 M NaCl stress treatment. (**C**): CAT content of wild-type and overexpression lines under 0.5 M Na_2_CO_3_ stress treatment. (**D**): CAT content of wild-type and overexpression lines under 0.5 M NaHCO_3_ stress treatment. ** *p* < 0.01, *** *p* < 0.001, standard error of three biological replicates.

**Figure 13:**
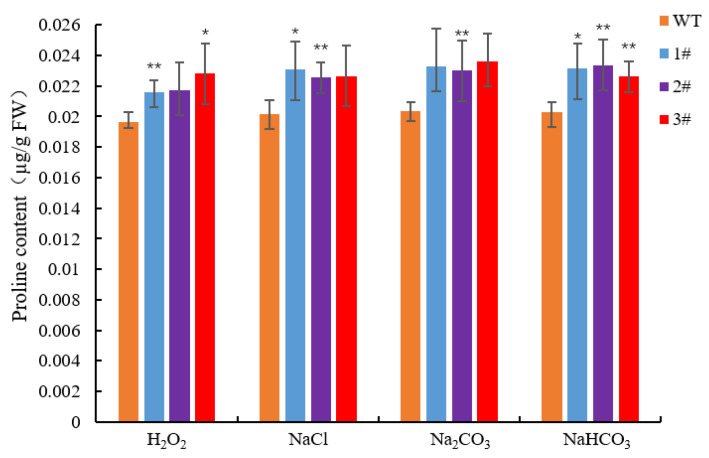
Proline content of *L. pumilum* under stress. Changes in proline content of WT and *LpGDSL* overexpression plants (1#, 2#, 3#) before and after 2 M H_2_O_2_, 0.6 M NaCl, 0.5 M Na_2_CO_3_, and 0.5 M NaHCO_3_ stress. * *p* < 0.05, ** *p* < 0.01, standard error of three biological replicates.

The results of the H_2_O_2_ content measurement showed that the content of the overexpressed lines was significantly lower than that of the wild-type lines after stress (Figure 11B). The CAT content of the overexpressed lines and the wild type, especially under NaCl stress treatment, could reach 1.63-fold (Figure 12), and the degree of CAT accumulation in *LpGDSL*-overexpressed *L. pumilum* was higher than that of the wild type. The proline content of the substrate that can be consumed to produce ROS was measured, and the results showed that the overexpressed lines could accumulate more proline, while the wild-type lines obviously accumulated a small amount of proline after stress (Figure 13). The results illustrated that the staining results of NBT and DAB showed no significant difference between the wild-type and overexpressed plants under the control condition (CK). The staining deepened after salt stress treatment, but the overexpressed leaves were lighter in color than the wild-type leaves, indicating that the overexpression of the *LpGDSL* gene reduced the accumulation of ROS in the plants under salt stress (Figure S6). 

**Figure 14:**
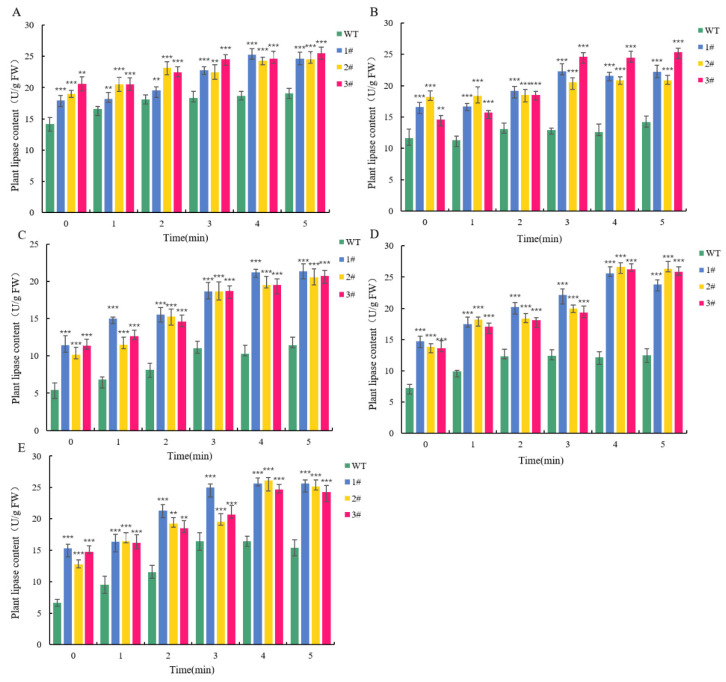
Plant lipase content of *L. pumilum* under stress. (**A**): Plant lipase content of wild-type and overexpression lines without any stress treatment. (**B**): Plant lipase content of wild-type and overexpression lines under 2 M H_2_O_2_ stress treatment. (**C**): Plant lipase content of wild-type and overexpression lines under 0.6 M NaCl stress treatment. (**D**): Plant lipase content of wild-type and overexpression lines under 0.5 M Na_2_CO_3_ stress treatment. (**E**): Plant lipase content of wild-type and overexpression lines under 0.5 M NaHCO_3_ stress treatment. ** *p* < 0.01, *** *p* < 0.001, standard error of three biological replicates.

Lipase activity in vivo was significantly reduced in wild-type lines after stress, and the content of lipase was greatly reduced within five minutes (Figure 14). 

The stems of the wild-type and *LpGDSL* overexpression lines were stained with cork Sudan III. Compared with the wild-type overexpression lines, the degree of cork formation was obvious, and cork mass accumulated in the vascular cambium of the plant (Figure S7A). Lignin staining was also performed. The xylem of the overexpressed lines was significantly thicker than that of the wild type (Figure S7B). The lignin content in the stems of all three overexpression lines (1#, 2#, 3#) was significantly higher than that of the WT (Figure S8). The *LpGDSL* may confer salinity resistance to the plants by increasing the lignin content.

### 2.5. Yeast Two-Hybrid Screening Interacting Proteins

**Figure 15:**
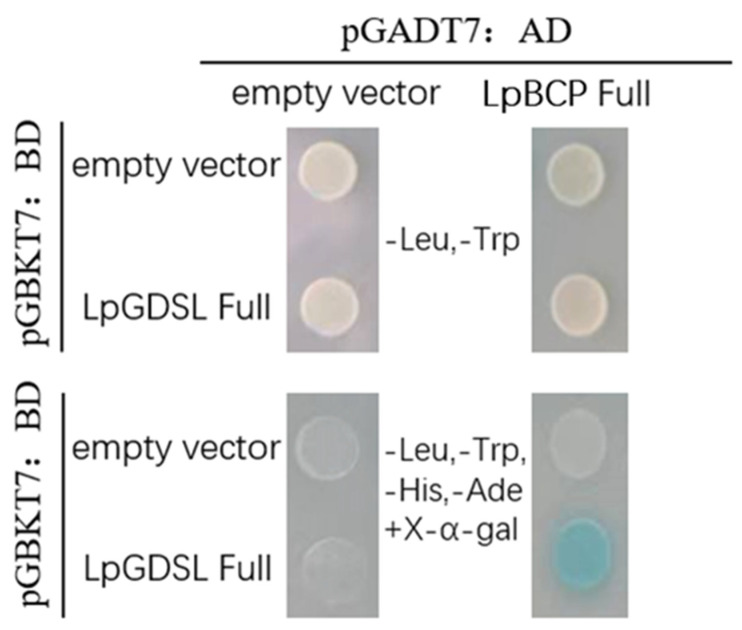
LpGDSL and LpBCP yeast two-hybrid verification co-transformation of pGADT7-*LpB3* and pGBKT7-*LpGDSL* recombinant plasmids into Y2H Gold strain. The pGADT7 + pGBKT7, pGADT7 + pGBKT7-*LpGDSL*, and pGBKT7 + pGADT7-*LpB3* co-transformed strains were used as controls. The first row of pictures shows the growth of the four strains on SD/-Trp-Leu medium, and it can be seen that all four colonies can grow; the second row of pictures shows the growth of the four strains on SD/-Trp-Leu-His-Ade + X-α-gal medium.

The cDNA library was used to screen the interacting proteins of LpGDSL, and the 12 sequences with successful sequencing results are listed in Table S3. We were surprised to find BCP genes included in the screened interactions proteins. The interaction between LpBCP and LpGDSL was verified by yeast two-hybrid and BiFC experiment. The control and all experimental groups cultured on SD/-Trp-Leu medium had normal yeast growth. However, only the pGBKT7-*LpGDSL* and pGADT7-*LpBCP* co-transformed strains could grow blue colonies on SD/-Trp-Leu-His-Ade + X-α-gal solid medium, while no blue growth trend was observed in the control group (Figure 15). 

**Figure 16:**
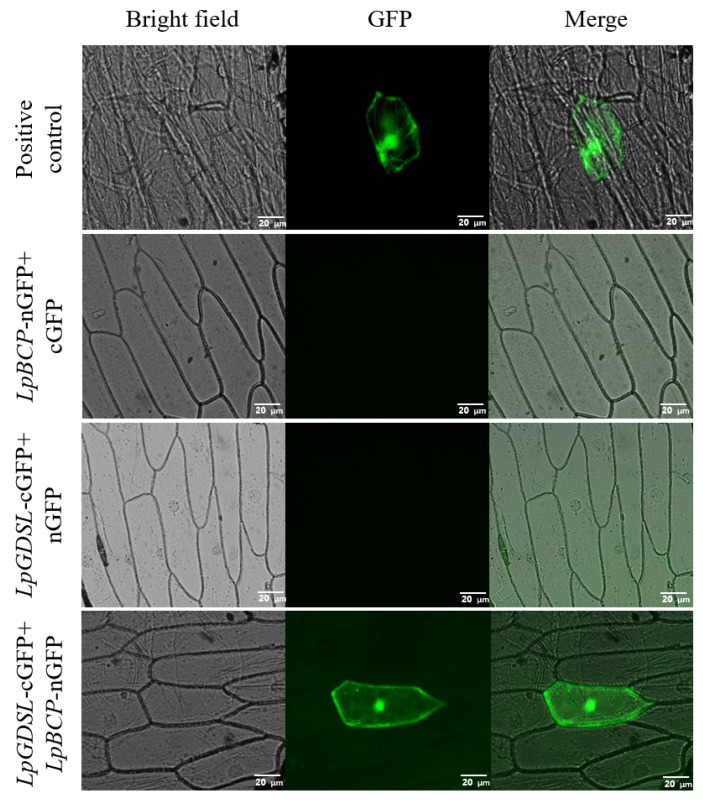
BiFC assays the interaction between LpGDSL and LpBCP. Co-transformation of pBS-35S: *LpGDSL*-VC80 and pBS-35S: *LpBCP*-VN154 plasmids into onion epidermis by gene gun method. pBS-35S: VC80 + pBS-35S: VN154 as positive control, pBS-35S: VC80 + pBS-35S: *LpBCP*-VN154 and pBS-35S: *LpGDSL*-VC80 + pBS-35S: VN154 as negative controls. Scale bar = 20 µm.

The constructed plasmids pBS-35S: *LpGDSL*-VC80 and pBS-35S: *LpBCP*-VN154 were transformed into onion skins by the gene gun method. pBS-35S: VC80 + pBS-35S: VN154, pBS-35S: VC80 + pBS-35S: *LpBCP*-VN154, and pBS-35S: *LpGDSL*-VC80 + pBS-35S: VN154 were used as the three control groups. As shown in Figure 16, fluorescence signals were observed in onion skins co-expressed with LpGDSL-cGFP and LpBCP-nGFP proteins, consistent with the positive controls, while no fluorescence was detected in the other two controls. The above results indicated the interaction between the LpGDSL and LpBCP proteins.

### 2.6. Promoter Cloning and Analysis

The promoter region of the *LpGDSL* gene was cloned and a 911 bp fragment was obtained successfully (Table S4). Plantcare software (https://bioinformatics.psb.ugent.be/webtools/plantcare/html/, accessed on 7 October 2023) was used to analyze and screen the active elements of the *LpGDSL* promoter region (Table S4), and then TBtools software V1.0 was used to map the selected active elements. The promoter region of *LpGDSL* contains many cis-acting elements related to saline–alkali resistance (Table S5). Notably, it also contains the acting site (CACCTG) of the B3 transcription factor. The position of the B3 transcription factor can also be seen in the figure of the acting element (Figure S9). Therefore, we selected the B3 transcription factor for subsequent experiments.

**Figure 17:**
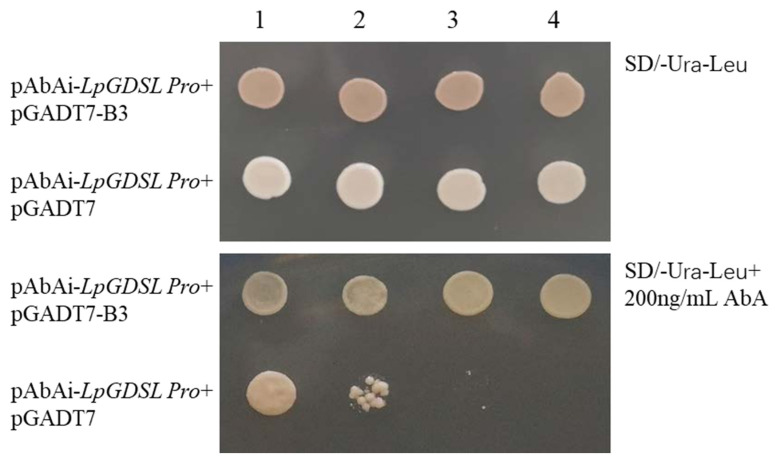
Yeast one-hybrid broth identification. The yeast strains co-transformed with pAbAi-*LpGDSL*-Pro and pGADT7-B3, pAbAi-*LpGDSL*-Pro, and pGADT7 null were diluted to 10, 100, 500, and 1000 times (keeping the initial bacterial concentration of all at OD_600_ = 0.5).

The growth of different bacterial liquid concentrations of the two strains were consistent on the SD/-Ura-Leu plate (Figure 17). On the SD/-Ura-Leu + 200 ng/mL AbA plate, the pAbAi-*LpGDSL* Pro + pGADT7-*LpB3* co-transfer could still maintain the normal growth trend at a certain concentration. This indicated that the B3 transcription factor could act on the *LpGDSL* promoter region. 

**Figure 18:**
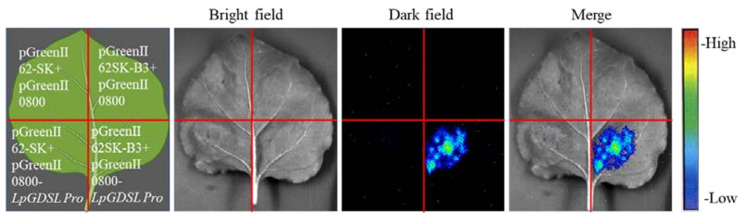
Dual-luciferase reporter assay. The pGreen II 62SK-B3 and pGreen II 0800-*LpGDSL* Pro recombinant plasmids were transfected into Agrobacterium, and tobacco with uniform growth was selected for injection. The pGreen II 62-SK + pGreen II 0800, pGreen II 62SK-B3 + pGreen II 0800, and pGreen II 62-SK + pGreen II 0800-*LpGDSL* Pro were the control groups.

To further verify the relationship, transcriptional activation tests were performed; pGreenII62-SK + pGreenII0800 and pGreenII62SK-*B3* + pGreenII0800 were co-injected with pGreenII62-SK + pGreenII0800*-LpGDSL* Pro as the control group. Fluorescence was detected only in the tobacco leaf region where pGreenII62SK-B3 + pGreenII0800-LpGDSL Pro was injected together (Figure 18), which verified that the B3 transcription factor could act on the promoter region of the *LpGDSL* gene to initiate downstream gene expression. 

## 3. Discussion

GDSL functions in plants have been found to include participation in plant growth and development, lipid metabolism, and stress resistance, and it is predicted that this protein may be localized in the whole cell to play a role [14]. 

The GDSL relationship with salt and alkali stress was investigated in this study. The *LpGDSL* gene was successfully cloned from the bulbs of *L. pumilum* (Table S1). LpGDSL has a highly conserved domain SGNH (Figure S1), which is a lipolytic enzyme. The LpGDSL protein has high homology with the GDSL protein of various plants (Figures S2 and S3). Among these plants, GDSL from *Dendrobium catenatum* (*DcGDSL*) has been found to play crucial roles in stress responses, plant growth, and development [15]. LpGDSL has the closest genetic relationship with DcGDSL, and LpGDSL may have similar functions to DcGDSL, which play crucial roles in stress responses.

In order to preliminarily verify whether the gene is related to saline stress, the overexpressing yeast were used to preliminarily validate this gene for saline resistance. On the stress medium containing 2.8 mM H_2_O_2_, 600 mM NaCl, 20 mM Na_2_CO_3_, and 22 mM NaHCO_3_, it was clearly observed that the pYES2-*LpGDSL* recombinant yeast grew faster compared to the empty vector yeasts (Figure 3). Through the analysis of the data obtained after qPCR, it was found that the *LpGDSL* gene was expressed in all tissue parts of *L. pumilum*, and its expression level reached the highest in leaves, followed by bulb and flower tissues (Figure 1). It was found that the expression of *LpGDSL* showed an up-regulation trend compared with the control group under various stresses (Figure 2), which proved that *LpGDSL* had an obvious response to saline–alkali stress, and it was preliminarily predicted to participate in the stress response of plants.

GDSL is a class of lipolytic enzymes and has lipase activity [16]. The lipase activity of LpGDSL was determined by the determination of protease activity (Figure 5). The resistance of the LpGDSL protein expressed in bacterial solution was inhibited under different stress conditions, but compared with the PGX-6P-3 vector, it showed an obvious tolerance to stress (Figure 6), which proved that the LpGDSL is a lipase gene that can respond to saline–alkali stress.

The GDSL family of OsGLIP genes may have potential roles in rice development and abiotic stress [17]. The same results were obtained in the in vivo experiments in plants, after successfully obtaining high expression of *LpGDSL*-overexpressing *L. pumilum* (Figure S4). It was found that the phenotypes of *L. pumilum* seedlings under certain concentrations of stress also showed that the overexpressing *L. pumilum* was more saline-tolerant (Figure 7 and Figure 8), and Western blot experiments on these seedlings revealed that the expression of the LpGDSL protein was increased after stress and was much higher than that in the wild type (Figure S5). Measurements of chlorophyll similarly showed better resistance in the *LpGDSL* overexpression lines (Figure 9). In order to ascertain the underlying mechanism responsible for the salinity tolerance observed in the *LpGDSL* overexpression lines, the malondialdehyde content was quantified (Figure 10), which was subjected to greater membrane oxidative damage compared to the wild type after the lower stress. The results of the DAB and NBT staining procedures indicated that the overexpression lines demonstrated superior ROS removal capabilities in the plants (Figure S6), which in turn led to the measurement of the indicators related to ROS. The overexpression strain demonstrated a reduction in superoxide anion and hydrogen peroxide content (Figure 11) and an increase in CAT and proline content (Figure 12 and Figure 13) in comparison to the wild type. This suggests that the *LpGDSL* overexpression lines are regulated to be subjected to saline and alkaline stress by means of balanced ROS. It was concluded from the results of lipase activity that salt and alkali stress greatly affected the lipase activity of lily lines, while the overexpression lines could well maintain the activity of plant lipase and maintain the plant under normal growth conditions (Figure 14). 

GDSL motif lipase/hydrolase family genes are involved in aliphatic suberin assembly [18]. Rice GDSL (DARX1) has a very important role in secondary wall formation. The lower cellulose content in *DARX1* mutants disrupts secondary wall formation and patterning and reduces mechanical strength [19]. Cotton *GhGDSL*, which exhibits secondary cell wall stage expression during spike development in cotton, plays an important role in secondary cell wall formation during spike development [20]. Experimental evidence shows that LpGDSL is also related to the lignin content. The *LpGDSL* overexpression lines were found to accumulate heavily in the vascular formation layer by corky Sudan III staining (Figure S7). The *LpGDSL* gene may be involved in the synthesis of cork in plants to improve the embolization of plant cells to form the cork layer, so as to isolate the internal tissues of plants, form a barrier to protect the internal tissues, and improve the saline–alkali resistance of plants. Additionally, the xylem of the overexpression lines was found to be significantly thicker than that of the wild type by lignin staining (Figure S8). In summary, *LpGDSL* can confer better salinity tolerance to plants and can better maintain the desired growth status of plants under stress injury compared to the wild type.

In order to further explore the functional mechanism of *LpGDSL* gene, 13 interacting proteins were screened by a yeast two-hybrid screen library, including the BCP. The BCP (blue copper protein) is a small protein with oxidizing activity. LpBCP greatly improves salinity stress tolerance in plants and the BCP was involved in the lignin metabolic pathway, affecting plant secondary metabolism and cell wall synthesis [21,22,23]. The AtBCB protein is involved in the oxidative stress response in plants. LpBCP increased NaHCO_3_ resistance by enhancing lignin or ROS scavenging in *Nicotiana benthamiana* [24].

The results of the yeast co-transformation (Figure 15) and BiFC (Figure 16) experiments further validated the interaction of the protein with the BCP. LpGDSL and the BCP may work together to confer salt tolerance to plants by enhancing lignin or ROS scavenging in *L. Pumilum*.

The B3 gene superfamily is mainly found in gymnosperms, mosses, green algae, and plants [25]. In plants, the B3 transcription factor can respond to growth and development, hormone signal transduction, and plant stress [26]. B3 has been verified as a transcription factor that can respond to saline–alkali stress [27]. *GhERF* is a member of the B3 family, and *GhERF* overexpression in *A. thaliana* exhibits enhanced salt tolerance and shows the enhanced regulation of the relevant biochemical parameters and the expression of genes involved in ROS scavenging [28]. 

Xylem-formation-related B3 genes were highly expressed in the differentiating xylem of six month-old *Populus alba* × *P. glandulosa* trees [29]. Tension wood development involves the transregulation of secondary cell wall genes—two significant transcription factor genes being B3 and MYB092—which leads to altered wood properties for stress adaptation [30].

The promoter region of *LpGDSL* was cloned using the chromosome walking method, and the acting elements were analyzed (Figure S9). It was preliminarily verified that the B3 transcription factor could act on the *LpGDSL* promoter region by the yeast monohybrid experiment method (Figure 17). In order to further verify the relationship between the B3 transcription factor and the *LpGDSL* promoter region, transcriptional activation activity was detected (Figure 18). The experimental results confirmed that the B3 transcription factor could indeed act on the *LPGDSL* promoter region. Therefore, we can show that the B3 transcription factor can act on the *LpGDSL* promoter to regulate downstream gene expression, allowing further exploration of the action mechanism of the *LpGDSL* gene.

Both the upstream transcription factor B3 and the interacting BCP can scavenge ROS and increase the content of lignin. Through experiments, it has been proven that *LpGDSL* also has the same function; LpGDSL scavenges ROS in order to maintain ROS homeostasis in the plant and reduce its damage (Figure 11, Figure 12 and Figure S6). LpGDSL also increases the lignin and cork content in plants under adverse conditions (Figures S7 and S8). Cork and lignin are major components of plant cell walls. The increased levels of lignin and cork increase the thickness of plant cell walls and improve salt tolerance in *L. pumilum*. 

**Figure 19:**
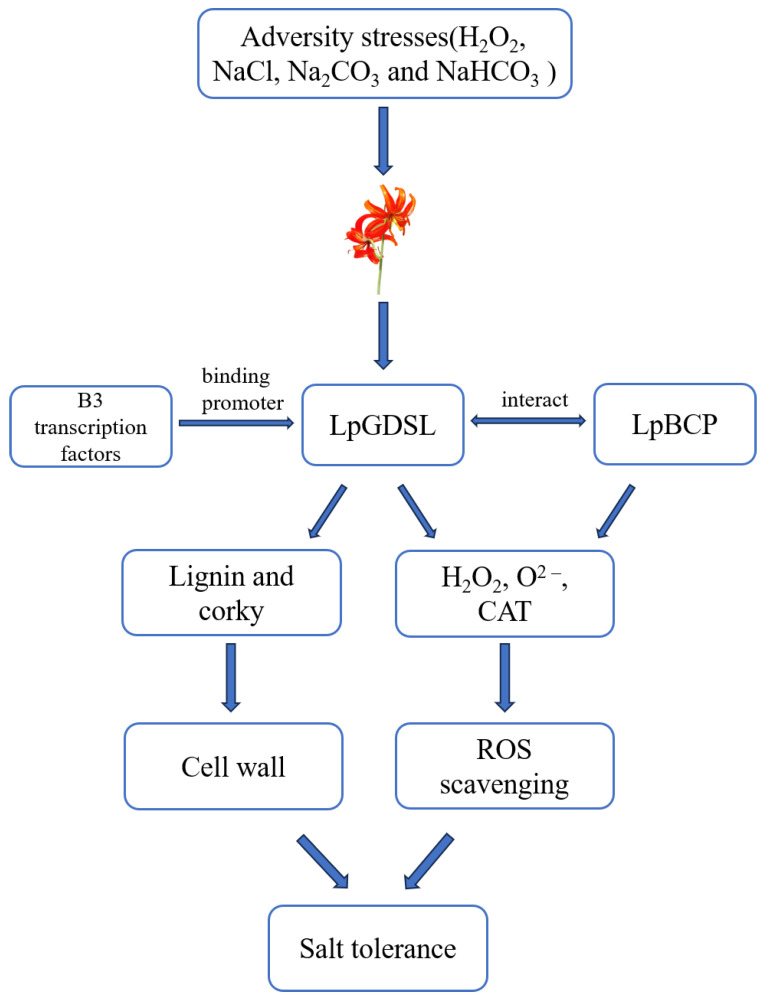
Model for LpGDSL-mediated response and tolerance to salinity stress. Under salt and alkali stress (NaCl, NaHCO_3_, Na_2_CO_3_, and H_2_O_2_), we suggest that LpGDSL may enhance the saline and alkali resistance of *L. pumilum*, mainly through the molecular pathway of ROS scavenging and by increasing the content of lignin. Accordingly, we hypothesized that the expression levels of B3, LpGDSL, and LpBCP would increase when *L. pumilum* was subjected to saline stress, that B3 acted as a transcription factor to initiate the expression of LpGDSL, and that LpGDSL and LpBCP interacted with each other, so that the three proteins could enhance the saline resistance of *L. pumilum* through the scavenging of ROS and accumulation of lignin in the plant. LpGDSL and LpBCP interacted with each other.

Considering the above, we conclude that *LpGDSL* may improve the saline–alkali resistance of *L. pumilum*, mainly through the molecular pathway of scavenging ROS and increasing the content of lignin. Based on this, we speculate that when *L. pumilum* encounters saline–alkali stress, the expression level of B3, *LpGDSL*, and *LpBCP* will be increased. B3 acts as a transcription factor to initiate the expression of *LpGDSL*, LpGDSL, and LpBCP to interact with each other. These three proteins work together to improve saline and alkali resistance in *L. pumilum* by scavenging ROS and accumulating lignin content in the plant (Figure 19).

## 4. Materials and Methods

### 4.1. Plant Materials and Growing Conditions

*L. pumilum* was collected from saline soil in Northeast China. The saline type is dominated by soda salinization. The groundwater and soil salt composition is dominated by carbonates. Saline soils have a pH of 8.46 [31]. Tobacco (*Nicotiana Benthamian*) seeds are kept by this laboratory. The *L. pumilum* and tobacco were cultured in a culture chamber at 25 °C under a photoperiod of 16 h light/8 h darkness.

### 4.2. Gene Cloning and Bioinformatics Analysis

Total RNA of *L. pumilum* was extracted by Trizol method [32]. The quality and concentration of the extracted RNA was detected by a BioSpec-Nano instrument (A11645200566, Shimadzu corporation, Shimane, Japan), and the cDNA was synthesized using a reverse transcription kit (Takara, Tokyo, Japan). The open-reading-frame sequence of the *LpGDSL* was found in the transcriptome sequencing results of the *L. pumilum* bulb. Specific primers *LpGDSL*F and *LpGDSL*R (shown in Table S2) were designed by SnapGene software 6.0.2. PCR amplification was performed using *L. pumilum* cDNA as template, and PCR products were sequenced and named *LpGDSL*. 

The GDSL with a higher homology than the other species was identified by BLAST analysis in NCBI. The amino acid sequence was compared by DNAMan software 9.0, and the conserved domain was analyzed by CD-Search. The phylogenetic evolutionary tree was constructed with MEGA 7 software V7.0.26.

### 4.3. LpGDSL mRNA Level Expression Specificity Analysis

Total RNA was extracted from flowers, leaves, bulbs, root tissues, and seeds of *L. pumilum* and reverse-transcribed into cDNA. The qPCR-specific primers (*LpGDSL*-qPCR-F and *LpGDSL*-qPCR-R, shown in Table S2) and cDNA templates were designed based on the sequence of the *LpGDSL* gene. *LpActin F* and *LpActin R* were used as a control, and Ultra SYBR Mixture was used as fluorescent dye.

*L. pumilum* with uniform growth was transferred into 1/2 MS medium containing 11 mM H_2_O_2_, 200 mM NaCl, 25 mM Na_2_CO_3_, and 20 mM NaHCO_3_ for 6 h, 12 h, 24 h, 36 h, and 48 h, respectively. RT-qPCR was conducted to observe the expression of *LpGDSL* in *L. pumilum* after stress treatment. Each sample was repeated three times.

### 4.4. Yeast Resistance Analysis

Using the *LpGDSL*-T plasmid as a template, the primers (*LpGDSL Bam*HI-F and *LpGDSL Xho*I-R, shown in Table S2) were designed, and these two sites were used to cut the yeast expression vector pYES2. *LpGDSL* was linked to the pYES2 yeast expression vector, and the successfully identified pYES2-LpGDSL plasmid was transferred into the yeast expression strain Inscv1 [33]. 

The strain of the pYES2-transformed yeast (as the control) and pYES2-LpGDSLwere incubated in fresh YPD liquid medium for 2 days at 30 °C 140 rpm with shaking. The obtained broth (OD_600_ = 0.6) was subsequently gradient-diluted (10^−1^, 10^−2^, 10^−3^, and 10^−4^) and 3 μL spots were cultured on the YPD solid medium under different stresses (2.8 mM H_2_O_2_, 600 mM NaCl, 20 mM Na_2_CO_3_, and 22 mM NaHCO_3_) for 3 days to analyze the yeast stress treatment. This was performed as follows: A volume of 3–4 µL of the aforementioned gradient-diluted yeast solution was pipetted onto the medium in a vertical orientation. The solution was then left for a period of 5 min to allow for evaporation, after which the plate was closed.

### 4.5. LpGDSL Protein-Induced Expression and Purification

*LpGDSL* was constructed into the pGEX-6P-3 protein expression vector (primer: *LpGDSL*-*BamH* I F and *LpGDSL*-*Xho* I R, shown in Table S2). 

The successfully constructed plasmid was transferred into the protein-expressing strain BL21. The pGEX-LpGDSL fusion protein was induced by different concentrations of IPTG (0, 0.5, 1, 1.5, and 2 mM) at 30 °C for 4 h, and the expression conditions were optimized; then, the fusion protein of pGEX-LpGDSL was purified [34]. LpGDSL protease activity was then determined. Using p-nitrophenyl acetate as the substrate, the purified protein content was ensured to be 2–4 µg. Then, after incubating at 30 °C for 60 min, the OD_405_ value was measured every 5 min, and the purified protein of the pGEX-6p-3 empty vector was used as the control group.

### 4.6. Acquisition and Resistance Analysis of Overexpressed Plants

*LpGDSL* was constructed into the PEH12 vector (primer: *LpGDSL*-F and, *LpGDSL*-R shown in Table S2), the recombinant LpEH12-LpGDSL plasmid was transferred into the Agrobacterium *EHA105* by electric shock method, and Agrobacterium *EHA105* with the LpEH12-LpGDSL plasmid was transferred into *L. pumilum* by the *agrobacterium tumefaciens* overexpression method [35]. The expression level of *LpGDSL* in overexpressed *L. pumilum* was determined by RT-qPCR with the wild type as the control, and the three plants with higher expression levels (#1, #2, and #3) were selected for follow-up experiments. 

The identified *LpGDSL*-overexpressing *L. pumilum* (#1, #2, #3), wild-type aseptic seedlings, and soil-grown seedlings were subjected to stress treatment, respectively. The aseptic seedlings were subjected to 20 mM H_2_O_2_, 200 mM NaCl, 10 mM Na_2_CO_3_, and 20 mM NaHCO_3_ solutions for stress treatment, and the soil-grown seedlings cultured at the same time were subjected to 2 M H_2_O_2_, 0.6 M NaCl, 0.5 M Na_2_CO_3_, and 0.5 M NaHCO_3_ for irrigation stress, and photographs were taken to observe the phenotypic changes. 

The proteins were extracted and subjected to Western blot [36] to characterize the protein change in the overexpression lines under stress compared to the wild type. Well-grown wild-type and *LpGDSL*-overexpressing *L. pumilum* of the same growth cycle were selected for protein extraction, and protein expression was detected by Western blot assay. Actin protein is a mouse monoclonal antibody (Abbkine, Shanghai, China).

Chlorophyll, malondialdehyde, superoxide anion, hydrogen peroxide, catalase, and proline contents, as well as the photosynthesis coefficient and plant lipase content activity were determined [21,37,38]. The stems of *L. pumilum* were then used for thrombosed staining and lignin staining, and the leaves were used for DAB and NBT staining. The nitro-blue tetrazolium (NBT) and diaminobenzidine (DAB) staining methods were utilized to detect O_2_^−^ and H_2_O_2_ in seedling leaves in situ, so as to understand the ability of LpGDSL to reduce ROS content in plants. The deeper the blue color is, the more O_2_^−^ that has accumulated. The darker brown the color is, the more H_2_O_2_ that has accumulated [21].

### 4.7. Screening and Validation of LpGDSL-Interacting Proteins by Yeast Two-Hybrid

The successfully constructed pGBKT7-*LpGDSL* (*LpGDSL EcoR*I F and *LpGDSL BamH*I R) was transformed into the Y2H Gold yeast strain. Interacting proteins were screened in cDNA library. The screened interacting protein LpBCP was constructed into the pGADT7 vector (*LpBCP EcoR*I F, *LpBCP BamH*I R), and the identified recombinant plasmids PGADT7-*B3* and pGBKT7-*LpGDSL* were co-transformed into the Y2H Gold yeast strain. It was cultured on SD/-Trp-Leu and SD/-Trp-Leu-His-Ade +X-α-gal + AbA solid medium. The proteins interacting with pGADT7-*LpGDSL* were screened from the *Lilium pumilum* yeast cDNA library. Yeast DNA was extracted and sequenced to obtain the interaction protein sequences.

### 4.8. Validation of LpGDSL-Interacting Proteins by BiFC

*LpGDSL* was cloned into the pBS-35S: VC80: NOS vectors (*LpGDSL*-*Kpn*I-F and *LpGDSL*-*Sal*I-R), and *LpBCP* sequences were cloned into the pBS-35S: VN154: NOS vectors (*LpBCP*-*Sma*I-F and *LpBCP*-*Spe*I-R). The constructed plasmids pBS-35S: LpGDSL-VC80 and pBS-35S: LpBCP-VN154 were transformed into onion skins by the gene gun method [39]. pBS-35S: VC80 + pBS-35S: VN154, pBS-35S: VC80 + pBS-35S: LpBCP-VN154, and pBS-35S: LpGDSL-VC80 + pBS-35S: VN154 were used as the three control groups. Incubation was at 28 °C for 48 h, followed by observation with a fluorescence microscope (Zeiss AxioImage.Z2, Carl Zeiss AG, Oberkochen, Germany).

### 4.9. Promoter Cloning 

The promoter region of the *LpGDSL* gene was cloned using Genome Walking Kit (Takara, Tokyo, Japan). The primers required were *LpGDSL Pro* SP1, *LpGDSL Pro* SP2, and *LpGDSL Pro* SP3, as shown in Table S2. The obtained promoter sequence is shown in Table S4. The cloned *LpGDSL* promoter sequence was analyzed by Plantcare software (http://bioinformatics.psb.ugent.be/webtools/plantcare/html/, accessed on 7 October 2023), and then the screened action elements were mapped by TBtools software V1.0. 

### 4.10. B3 Regulation of the LpGDSL Promoter Validated by Dual-Luciferase Reporter 

B3 was constructed into the pGreen II 62SK vector (*LpB3 BamH*I-F and *LpB3 Xho*I-R, shown in Table S2), the *LpGDSL* promoter was constructed into the pGreen II 0800 vector (*LpGDSL* Pro *Xho*I-F and *LpGDSL* Pro *BamH*I-R), and the two recombinant plasmids were separately transfected into *Agrobacterium tumefaciens* GV3101 by freeze-thawing [40], followed by injection into tobacco leaves [41]. After dark culture for 2 days, luciferase was injected at the injection site and reacted for 5 min in the dark. Tobacco leaves were observed and photographed by a chemiluminescence imager (Tanon-4600SF, Shanghai Tianneng Technology Co., Ltd., Shanghai, China).

### 4.11. B3 Regulation of the LpGDSL Promoter Validated by Yeast One-Hybrid

The *LpGDSL* promoter sequence was constructed into the pAbAi vector (*LpGDSL* Pro *Hind*III F and *LpGDSL* Pro *XhoI* R), the *LpGDSL-*pAbAi recombinant plasmid was linearized by digestion with BstB1 and transformed into the Y1H Gold yeast strain, and the Y1H Gold transfected with the linearized *LpGDSL-*pAbAi recombinant plasmid was cultured on SD/-Ura medium for 3 days. The monoclonal strains were identified by PCR. The constructed recombinant plasmid pGADT7-B3 and the pGADT7 empty vector were transferred into the above prepared yeast receptor state and cultured in the solid medium of SD/-Ura-Leu, respectively. pAbAi-*LpGDSL* Pro + pGADT7 was used as a negative control. Then, 3–4 µL of the above bacterial solution was placed on an SD/-Ura-Leu + 200 ng/mL AbA plate and SD/-Ura-Leu plate, respectively, as follows: 1: 10-fold dilution; 2: 100-fold dilution; 3: 500-fold dilution; 4: 1000-fold dilution.

### 4.12. Statistical Analysis

The data processing for qPCR was conducted using MxPro-QPCR software v4.1. The software is found on the website https://www.manualslib.com/manual/1418060/Agilent-Technologies-Mx3000p.html (accessed on 10 July 2024). The significance of the difference analyses for all histograms was determined using SPSS 17.0 software. 

## 5. Conclusions

The open reading frame (ORF) of the gene *LpGDSL* was successfully cloned from *L. pumilum* with a length of 1080 bp. LpGDSL is closely related to the GDSL proteins of asparagus, date palm, and small-fruited wild plantain. The highest expression of *LpGDSL* was found in the leaves of *L. pumilum* by qPCR. *LpGDSL* was transformed into *L. pumilum* to obtain overexpression plants. *LpGDSL* improved plant salt tolerance according to the phenotypic analysis. Chlorophyll content, MDA content, O_2_^−^ content, H_2_O_2_ content, CAT content, proline content, plant lipase content, DAB and NBT staining, and lignin content revealed that the *LpGDSL* overexpression lines improved plant salinity resistance by scavenging ROS and increasing plant cell wall thickness. The LpGDSL protein interacting with LpBCP was detected by a yeast two-hybrid system. The *LpGDSL* promoter was cloned to analyze and verify that the B3 transcription factor initiates *LpGDSL* expression.

## Data Availability

Data is contained within the article and Supplementary Materials.

## Supplementary Materials

The following supporting information can be downloaded at: https://www.mdpi.com/article/10.3390/ijms25179319/s1.
