# Supplementary material for: GDSL in Lilium pumilum (LpGDSL) Confers Saline–Alkali Resistance to the Plant by Enhancing the Lignin Content and Balancing the ROS
Source: Int J Mol Sci. 2024 Aug 28;25(17):9319. doi: 10.3390/ijms25179319 (PMC11395047; doi:10.3390/ijms25179319)
Supplement: Supplementary file 1 [file ijms-25-09319-s001.zip › ijms-3125670-supplementary.pdf]

Table S1. *LpGDSL* open reading frame sequence

| name | sequence |
|---|---|
| The open reading frame sequence of *LpGDSL* | ATGGTCAGCACTGGATTTATTGTTCATCATGTGGTG<br>GTGGCTGCGGCTGCATTAGTCCTCATCTTGTCTGCA<br>CTTCCCAGCGGAAGTGCACAGCCACTCGTTCCGGC<br>CATCATCACATTTGGCGACTCAGCAGTCGATGTGGG<br>AAACAATGACTACCTCCCTACCCTCTTCAAAGCTGA<br>TTTCCCTCCTTATGGGCGGATTACAAAAACCATCA<br>AGCCACCGGGAGGTTCTGTAACGGGAAATTGGCTA<br>CTGATATCACTGCTGAAACTCTGGGATTCACGAGCG<br>TCCCCCCGCCATACCTCAGCCCACAGGCCTCGGGAA<br>AGAACCTTCTCATAGGAGCTAATTTTGCTTCAGCTGC<br>CTCTGGGTATTATGATGAAACTGCCATTTTGTATAAAG<br>CCATCCCATTATCTCAACAGTTGGAGTACTACAAAGA<br>GTACCAGTCCAAGTTGAAGTCTGTTGCTGGGAGTGG<br>GAAGGCGACCTCCATCCTATCAGGGGCGCTGTACATC<br>ATTGGCGCTGGGAGCAGCGACTTCATACAAAACTACT<br>ATGTCAATCCATACCTCAACAAGATCTACAGCCCCGA<br>TGAGTACTCTTCCTTCCTTGTCAGCATCTTCTCAAATT<br>TTGTCAAGGACCTATACGGCCTTGGGGCTCGAAGGAT<br>TGGAGTGACATCACTGCCGCCATTGGGGTGCCTCCCC<br>TTATCCAAAAATATCTTTGGTTACGGTAAAAGTGGCTG<br>TGTTGCGAACTTCAATGCTGATGCCAGAAACTTCAAC<br>AAGAAGGTCACGGCAGCGATCGACTCACTCACTAAG<br>AAGTTTCCAAACCTTAAGATCGCTGCCCTCGACATCTA<br>CACGCCTCTTCTCGAACTAGCCACCAATCCATCAAAAA<br>ACGGTTTCTTTGAGGCGGGGAGAGGCTGCTGCGGGAC<br>AGGGACGGTGGAGACGACATCGTTCCTCTGCAACCCC<br>AAGTCGCTCGGGACATGCTCAAACGCAACCGGCTACG<br>TGTTCTGGGACAGTGTGCACCCATCCCAAGCTGCAAAC<br>CAGTTTATCGCGGATTCGCTCATCCTTGCAGGCATTTCC<br>CTCGTTTCGTGA |

Table S2. Primers were used in this study

| Primer Name | Primer Sequence |
|---|---|
| *LpGDSLF* | ATGGTCAGCACTGGATTT |
| *LpGDSLR* | TCACGAAACGAGGGAAAT |
| *LpGDSL B*amH I *F* | ggatccATGGTCAGCACTGGAT |
| *LpGDSL X*ho I *R* | ctcgagTCACGAAACGAGGGAA |
| *LpGDSL* qPCR *F* | TTAAGATCGCTGCCCTCGAC |
| *LpGDSL* qPCR *R* | CACGAAACGAGGGAAATGCC |
| *LpActin F* | GCATCACACCTTCTACAACG |
| *LpActin R* | GAAGAGCATAACCCTCATAGA |
| *LpGDSL Eco*R I *F* | gaattcATGGTCAGCACTGGATTT |
| *LpGDSL Bam*H I *R* | ggatccTCACGAAACGAGGGAAAT |
| *LpBCP F* | ATGGCTGGTGCTGTAGT |
| *LpBCP R* | TTAGCCAAATAATGCAA |
| *LpBCP Eco*R I *F* | gaattcATGGCTGGTGCTGTA |
| *LpBCP Bam*H I *R* | ggatccTTAGCCAAATAATGC |

| | |
|---|---|
| *LpGDSL Pro* SP1 | AAGGTTCTTTCCCGAGGCCTGT |
| *LpGDSL Pro* SP2 | TTTCCCGTTACAGAACCTCCCG |
| *LpGDSL Pro* SP3 | GCCAAATGTGATGATGGCCGGA |
| *LpGDSL Pro* F | CACATTGATGCCTGACTCAC |
| *LpGDSL Pro* R | CTTGGTTCTCTGGCACAAAC |
| *LpGDSL Pro Hind*Ⅲ F | AAGCTTCACATTGATGCCTGACTCAC |
| *LpGDSL Pro Xho*Ⅰ R | CTCGAGCTTGGTTCTCTGGCACAAAC |
| *LpB3 Bam*HⅠF | ggatccTCGCAAGTATGCCTGGTT |
| *LpB3 Xho*Ⅰ R | ctcgagATTCGAGACCCTCAACTC |
| *LpGDSL Pro Xho*Ⅰ *F* | ctcgagGCCTTAATTAACCAACTGCA |
| *LpGDSL Pro Bam*HⅠ R | ggatccCTTGGTTCTCTGGCACAA |
| *LpGDSL*-KpnI-F | ggtaccATGGTCAGCACTGG |
| *LpGDSL*-SalI-R | gtcgacTCACGAAACGAGGG |
| *LpBCP*-SmaI-F | cccgggATGGCTGGTGCTGT |
| *LpBCP*-SpeI-R | actagtTTAGCCAAATAATGC |

Table S3 Candidate interacting proteins obtained by yeast two-hybrid screening of *LpGDSL*

| serial number | gene name | gene annotation |
|---|---|---|
| 1 | SYP23 | Syntaxin of plants, key proteins that regulate vesicle transport (Ibrahim et al., 2020) |
| 2 | PHB3 | antiproliferative protein Prohibitin3 (Li et al., 2022) |
| 3 | CP47 | Photosystem II CP47 reaction center protein (Bečková et al., 2022); One of the components of the core complex of photosystem II (PSII). It binds chlorophyll and helps catalyze the primary light- induced photochemical processes of PSII. |
| 4 | IGPD | Imidazole glycerol-phosphate dehydratase; Belongs to the imidazole glycerol-phosphate dehydratase family (Qu et al., 2021). |
| 5 | HISN6 | Histidinol-phosphate aminotransferase (Rutkiewicz et al., 2023) |
| 6 | PMM2 | Phosphomannomutase (Zhang et al., 2016) |
| 7 | BRISC and BRCA1-A | Multifunctional Molecular Machines for Ubiquitin Signaling (Rabl, 2020) |
| 8 | EARLI 1 | Early Arabidopsis aluminum induced 1, also a member of the lipid transfer protein family (Feng et al., 2014). |
| 9 | WVD2 | Wave-dampened 2, a novel microtubule-associated protein (Perrin et al., 2007) |
| 10 | COBRA | A family of glycosyl-phosphatidyl inositol (GPI) anchoring proteins composed of COBL proteins family of anchoring proteins (Zhang et al., 2023) |
| 11 | BAH1 | E3 ubiquitin-protein ligase (Xu et al., 2018) |
| 12 | GMP | GDP-mannose pyro phosphorylase (Fu et al., 2023) |

Table S4 Sequence of *LpGDSL* promoter

| name | sequence |
|---|---|
| *LpGDSL* promoter sequence | GCCTTAATTAACCAACTGCATACTAGATAGGTAATA TATGGTAAAGTAGATTAGAAACACCTTAGATAATTT CTTTATTATCAATTTTGATTATATACTCTGATTTCAC CTTTTTAGTACTGACCCGTGCGCTGCGGACTAGCCA GCCAACCCCATGCATGCTTTTGTTTGAGAAGGTACC TGGCTTAATCTAACAGGCAACAGCTAATCCACATTA CATCAGTCCATCAACCTGCACAAATTGCTGGCTCGC TGTATAATCTTGTTCACAGAATTGTCTATGTGACCTT TGCCCTTCACCGCTCAAGTCCCGGTACAAGTACCCT TTTCCATCTACCAACAACTACTACATTAATTATATGT TAGAATTGTTTCACTGCCACTTATTTTAGGAGATTAT CGACACAGAAAATTGGAGTACGAAACAATTTTTCTT TCTTTCCGCCTTAACCACGGCTCACTGCTGAATAGCC CTATCCATCTCTCTGGAGTTGGTCCAATCTATATTTA GACTATTAAAATGTAAGCAAGAGGAGAACTACTTA ACCTTCTGTACCACTTTCCCCTTCATATGAGGAACAA TGAGGCAGCTGGAGTTTCCATTTAATGGAAAAGGCA GCCCGGTGTTCCTTGTAATGATATGTCCACTGTATTG CTCTTCCCAACCACCCTGCCATATCAATAAACCTAA TAAAGAGCCACCTTAGTTGTTGTAAGGATTGATGGA CAATGTGGGATGGATCTCTGCTTGTGCTAGCTTTTTA AAAGTTTGCCCACATGTATGCCTTCAATGCTCTTTAA CAAACCCCTTAGACTTTCCTAACAATCACCTCCATAT ATATGGGTCTATCCTCCCAGTAGAGCTTGTACCTGT AAAGCTGGCATTGTTTGTGCCAGAGAACCAAG |

Table S5 Analysis of Promoter Region Action Elements in *LpGDSL*

| cis-element | Motif (5'-3') | Function | Frequency |
|---|---|---|---|
| CAAT box | CAA | Regulates the transcription initiation of downstream genes and frequency | 19 |
| MYC | CATGTG | MYC binding site | 1 |
| MRE | AACCTAA | MYB binding sites involved in light response | 1 |
| GARE-motif | TCTGTTG | Gibberellin response element | 1 |
| Box 4 | ATTAAT | Conserved DNA modules involved in light response part of the conserved DNA module involved in light response | 1 |
| STRE | AGGGG | Osmotic stress response element | 2 |
| RY-element | CATGCATG | Cis-acting elements involved in seed-specific regulation | 1 |
| Myb-binding site | CAACAG | Myb binding site | 1 |

| | | | |
|---|---|---|---|
| GCN4_motif | TGAGTCA | Cis-regulatory elements involved in endosperm expression | 1 |
| CAT-box | GCCACT | Cis-acting elements associated with phloem tissue expression | 1 |
| ABRE | CGCACGTGTC | Cis-actions involved in the abscisic acid reaction component | 1 |
| GATA-motif | GATAGGG | Part of a light-responsive element | 1 |
| MYB | CAACAGTAACCA CAACCA | MYB binding site | 3 |
| P-box | CAACAAACCCCT T | Gibberellin response element and light response element | 1 |
| GT1-motif | GGTTAA | Components for Light Response | 2 |
| LAMP-element | CTTTATCA | Part of a light-responsive element | 1 |
| RAV1AAT | CAACA | The AP2 and B3 sample domains of RAV1 are connected through a highly flexible structure connecting this highly flexible structural linkage, allowing these two domains to interact with the CAACA and CACCTG motifs in a variety of spacings and CAACA and CACCTG motifs in various spacings and orientations. sequences. | 1 |

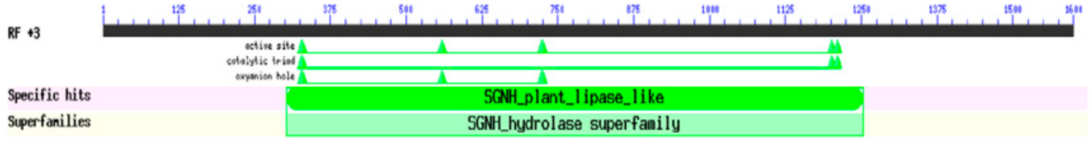


Figure S1. The conserved domain of LpGDSL protein. Predictive analysis of conserved structural domains of LpGDSL proteins using the Conserved Domain Search function on the NCBI website. The results showed the presence of a highly conserved structural domain SGNH phytolipase at position 300-1165 amino acids, which belongs to the SGNH hydrolase

superfamily.

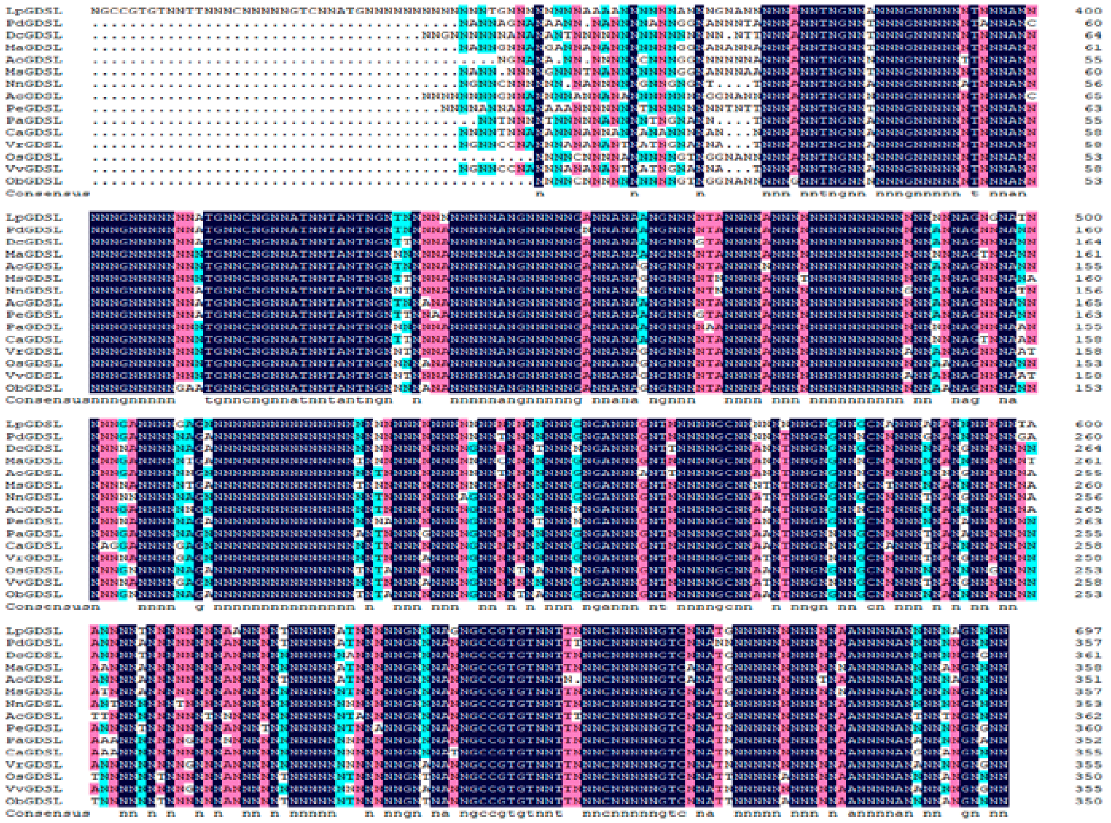

Figure S2. Amino acid sequence alignment of LpGDSL with homologous proteins. The amino acid sequences of GDSL cloned from lily of the valley were compared with those of GDSL proteins from other species by using DNA Man software. The dark blue indicates the highly conserved region of the sequence, the light blue indicates the similar region of the sequence, and the pink indicates the more similar region of the sequence.

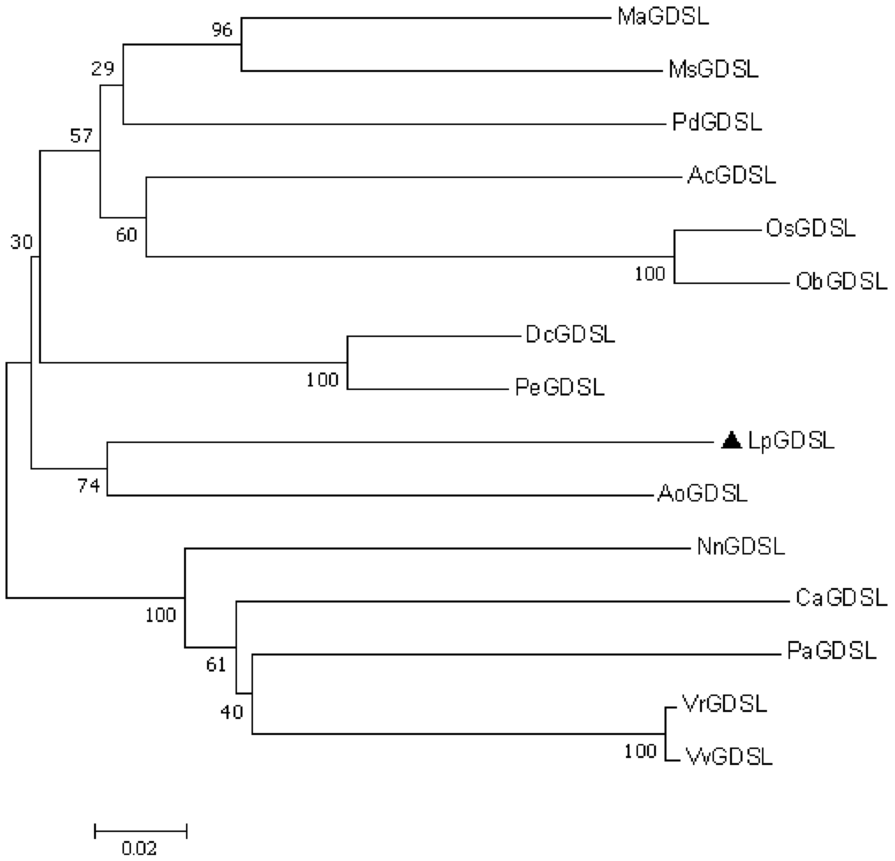


Figure S3. Phylogenetic tree analysis of LpGDSL. The MEGA7 program was used for the construction of phylogenetic trees.

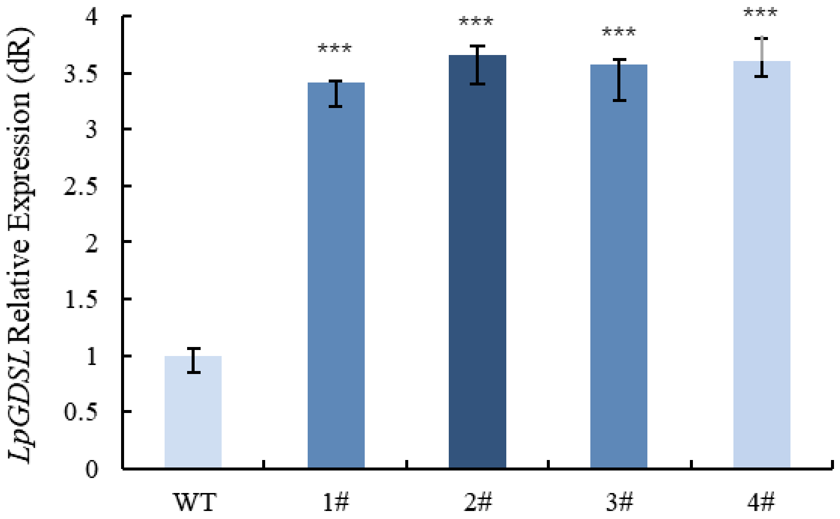


Figure S4. Identification of *L. pumilum* overexpressing *LpGDSL*. Leaf tissues of wild-type and transgenic *L. pumilum* were selected, and their cDNAs were used as templates for real-time

fluorescence quantitative PCR assay to observe the expression levels of *LpGDSL* in wild-type and four transgenic plants. "*" indicates significant difference, Student's *t* test ($P < 0.05$, ± SEM); "**" indicates extremely significant difference, Student's *t* test ($0.01 < P < 0.05$, ± SEM); "***" indicates extremely significant difference, Student's *t* test ($P < 0.01$, ± SEM).

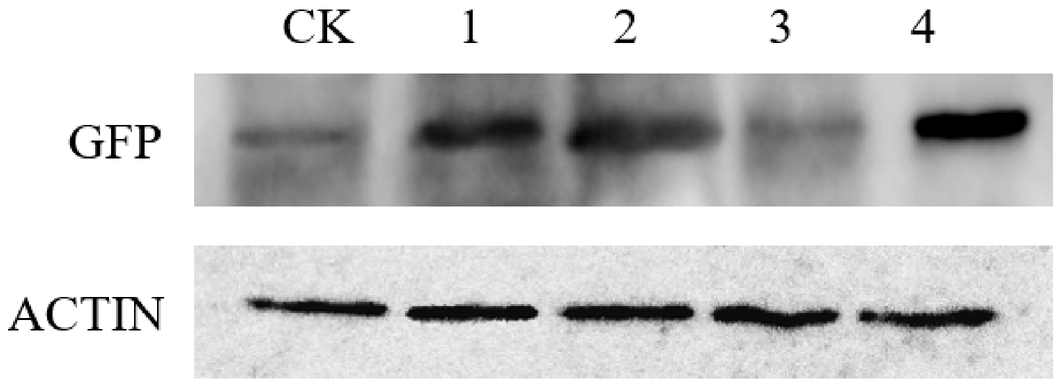


Figure S5. Western-blot of *L. pumilum* overexpressing *LpGDSL* under stresses. CK: Wild type control; 1: Under the stress treatment of 2 M $H_2O_2$; 2: Under the stress treatment of 0.6 M NaCl; 3: Under the stress treatment of 0.5 M $Na_2CO_3$; 4: Under the stress treatment of 0.5 M $NaHCO_3$.

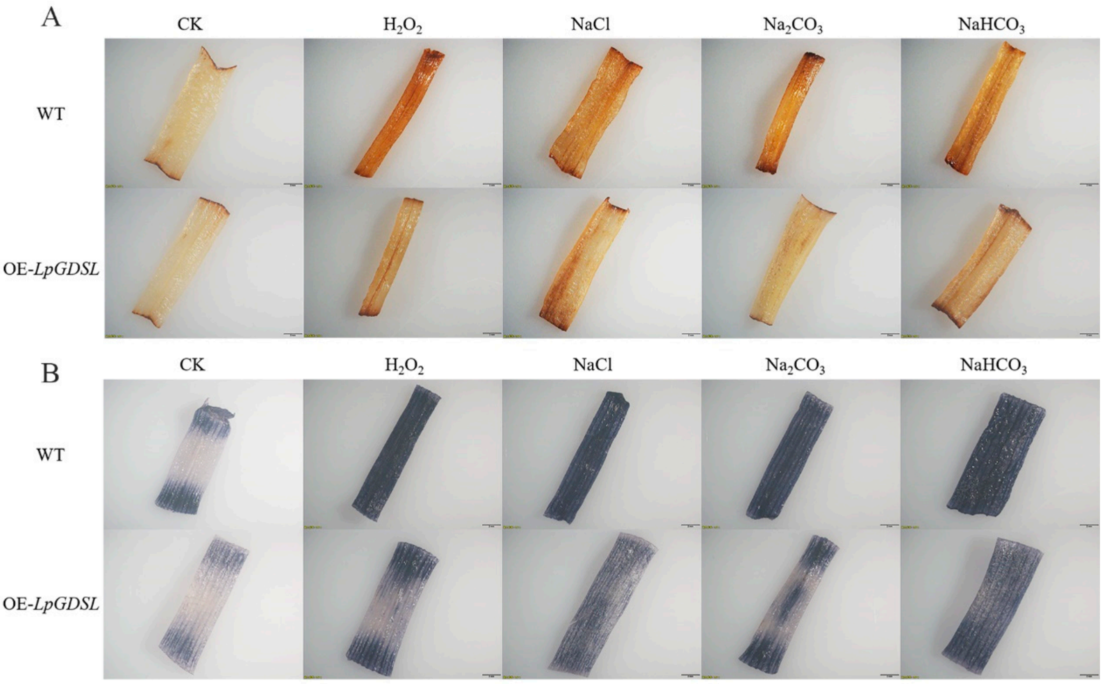


Figure S6. DAB and NBT staining of wild-type and overexpression plants under stress. After irrigation stress with 2 M $H_2O_2$, 0.6 M NaCl, 0.5 M $Na_2CO_3$, and 0.5 M $NaHCO_3$, the same parts of wild-type (WT) and overexpression *L. pumilum* (OE-*LpGDSL*) leaf were cut and stained with DAB and NBT, respectively. In situ NBT and DAB staining methods were used to detect the accumulation of $O_2^-$ and $H_2O_2$ in seedling leaves, which reflected the ability of LpGDSL to reduce ROS content in plants. The deeper the blue color is, the more $O_2^-$ accumulated. The darker the brown color is, the more $H_2O_2$ is accumulated. Under normal culture conditions (CK), both wild-type and overexpression plants were lightly colored. Both wild-type and overexpression plants became darker in color after stress treatment. However, the overexpression plants were significantly lighter in color than the wild-type plants, indicating that the ac-cumulation of $O_2^-$ and $H_2O_2$ in the overexpression plants was significantly lower than that in the wild-type.CK: No treatment has been done. Scale bar = 200 μm.

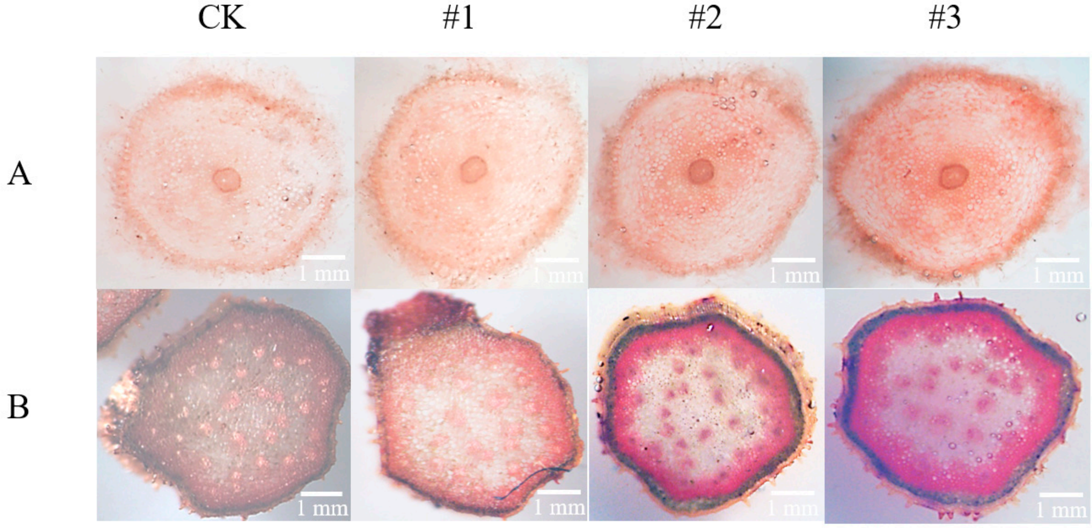


Figure S7. A. Thrombosed staining of *LpGDSL* transgenic *L. pumilum*. Stem corkiness of wild-type and overexpressed *LpGDSL L.Pumilum* stained for Sudan III. B. Lignification of *LpGDSL* transgenic *L. pumilum*. Lignin staining of stems of wild-type and overexpressed *LpGDSL L.Pumilum*. CK: Wild-type. *L.Pumilum*. #1, #2, #3: overexpressed *L. pumilum*. Do 3 repetitions of the over expression. Scale bar = 1 mm.

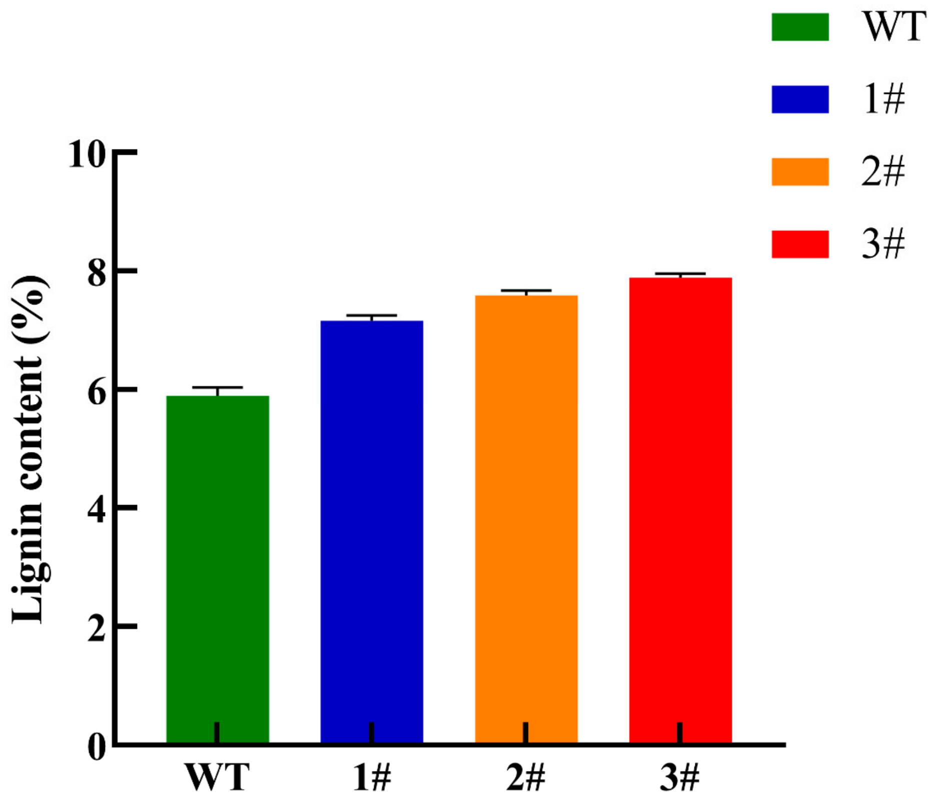


Figure S8. Lignin content in stems of WT and *LpGDSL* overexpressing plants. Lignin content was determined by UV spectrophotometry. Wild-type and overexpression plants with uniform growth were selected as experimental materials. Do three biological replicates. WT: Wild-type. #1, #2, #3: overexpressed *L. pumilum*.

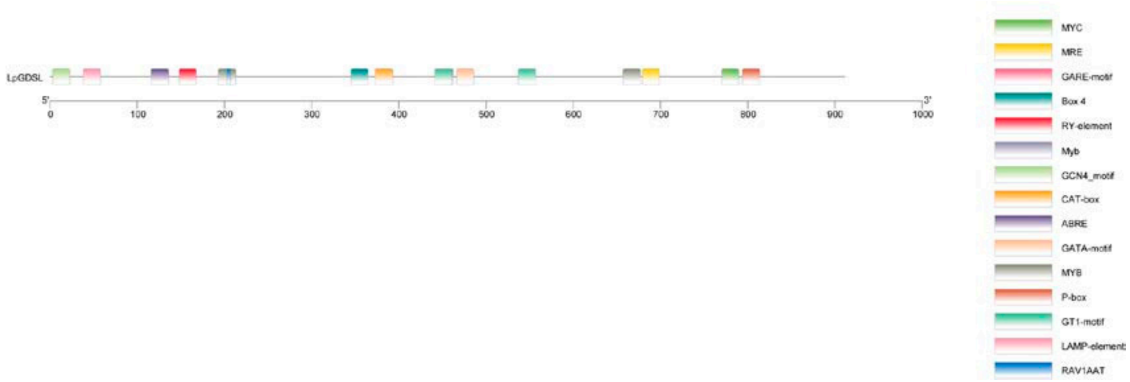


Figure S9. Analysis of Promoter Region Action Elements in *LpGDSL*.

Plant care software was used to analyses the promoter region of *LpGDSL* for its action elements.

TBtools software was used to map the screened actors.
